# Lavender compounds interfere with AI-2 dependent bioluminescence in *Vibrio harveyi* without affecting LuxS signaling in *Campylobacter jejuni*

**DOI:** 10.1016/j.isci.2026.115283

**Published:** 2026-03-07

**Authors:** Blaž Jug, Dina Jug, Sonja Smole Možina, Anja Klančnik

**Affiliations:** 1 Department of Food Science and Technology, Biotechnical Faculty, University of Ljubljana, Ljubljana, Slovenia

**Keywords:** microbiology, bacteriology

## Abstract

Bacterial intercellular communication is essential for biofilm formation. The interspecies signaling molecule autoinducer-2 (AI-2), produced by LuxS, is involved in *Campylobacter jejuni* intercellular signaling, metabolism, motility, virulence, and biofilm formation, although its receptor remains unidentified. We studied the effects of lavender preparations (essential oil and ethanol extracts of *Lavandula angustifolia*) and pure compounds (linalool and linalyl acetate) on the signaling mechanisms in *C. jejuni.* Using the *Vibrio harveyi* MM30 biosensor, we observed a significant reduction in bioluminescence following treatment with lavender-derived compounds. A *C. jejuni* 11168*ΔluxS* mutant was included as a negative control to distinguish effects on AI-2 production from interference with biosensor detection. AI-2 concentrations measured by HPLC-FLD remained unchanged. In addition, *luxS* expression (qPCR) and LuxS protein abundance (orbitrap-based mass spectrometry) were unaffected. The decreased bioluminescent response despite unaltered AI-2 concentrations indicates interference with AI-2 detection rather than production, a hypothesis further explored *in silico*. These findings highlight the complexity of AI-2 signaling and suggest lavender compounds as potential modulators of bacterial communication.

## Highlights

• Lavender-treated *C. jejuni* spent growth media reduce AI-2-dependent bioluminescence
• Lavender preparations do not alter AI-2 levels or *luxS* expression in *C. jejuni*
• *In silico* analyses support potential interference with AI-2 recognition in *V. harveyi*
• Lavender compounds reveal AI-2 independent mechanisms affecting cell communication

## Graphical abstract

**Figure:**
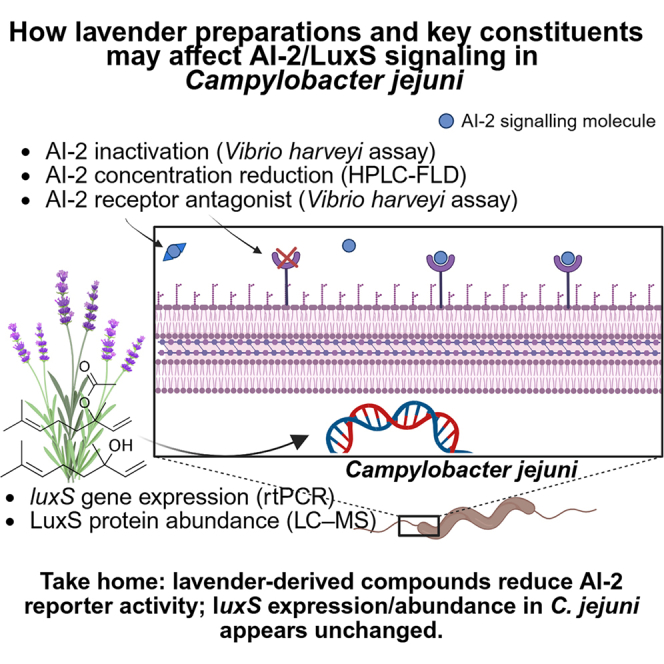

## Introduction

*Campylobacter jejuni* and *Campylobacter coli* are the primary culprits of gastroenteritis globally and the leading foodborne gastrointestinal pathogens in the European Union.^1^ They cause campylobacteriosis, which typically presents as diarrhea but can also lead to serious post-infection complications such as Guillain-Barré syndrome and reactive arthritis.^1^^,^^2^ Combating *Campylobacter* is challenging, especially because of its resistance to ciprofloxacin and tetracycline.^1^^,^^3^ *C. jejuni* exhibits great adaptability and resilience and is thus widely distributed, including in food production environments, such as farms and slaughterhouses.^4^^,^^5^

The ability of *Campylobacter* to remain infectious under various conditions highlights its zoonotic transmission potential through contaminated sources, such as unpasteurized dairy products, undercooked poultry, and water. Additionally, the ability of *Campylobacter* to form biofilms and survive in diverse environments is a key factor that enables its spread along the food production chain.^6^^,^^7^ This underlines the imperative for continuous monitoring and the development of novel control strategies targeting biofilms or their key processes, such as intercellular signaling.

Bacterial intercellular communication regulates virulence, nutrient acquisition, and biofilm formation, which are essential for bacterial survival, pathogenicity, and adaptation to the environment. Inter- and intracellular communication predominantly relies on N-acyl-homoserine lactones and autoinducing peptides in Gram-negative and Gram-positive bacteria, respectively. Additionally, both bacterial types recognize the universal signaling molecule autoinducer-2 (AI-2), which facilitates inter-species communication.^8^^,^^9^ AI-2 can be detected through various mechanisms, including the two-component signaling system LuxPQ, the LsrB family, and chemoreceptors.^10^ In *C. jejuni*, AI-2 is mediated by the *luxS* gene; however, its specific receptor remains unidentified despite extensive research.^11^ This partial AI-2 signaling system regulates motility, attachment to surfaces, biofilm formation, chemotaxis, and virulence.^12^^,^^13^^,^^14^^,^^15^^,^^16^^,^^17^

Recent research has revealed the significant effects of *luxS* on vital metabolic pathways in *C. jejuni*. Transcriptome and proteome analyses of *C. jejuni* NCTC 11168 showed that *luxS* deletion upregulates metabolic pathways critical for energy production, growth, and stress responses (e.g., oxidative phosphorylation, carbon metabolism, the citrate cycle, and the biosynthesis of secondary metabolites and essential amino acids). This metabolic adaptation enables better survival of the *luxS*-mutant under starvation, indicating that *luxS* plays a broader role than previously assumed, extending beyond intercellular signaling, motility, biofilm formation, and virulence.^18^

Previous studies have demonstrated the efficacy of various natural compounds in hindering intercellular signaling as an effective alternative approach to combating antibiotic-resistant strains without promoting further drug resistance. Such impaired intercellular signaling leads to decreased motility, adherence, virulence, and biofilm formation.^19^^,^^20^^,^^21^^,^^22^ Decanoic and lauric acids significantly inhibit AI-2 signaling and decrease biofilm formation in *C. jejuni*.^23^ Ethanol extracts from *Euodia ruticarpa* fruits and lavender preparations inhibit adhesion and biofilm formation in *C. jejuni*.^19^^,^^24^ A correlation has been observed between the decreased bioluminescent response of the *Vibrio harveyi* reporter strain (used to detect AI-2 in spent growth media (SGM) of *C. jejuni* treated with subinhibitory concentrations of plant preparations) and decreased motility and attachment of *C. jejuni* to abiotic surfaces.^22^ However, it remains unclear whether these plant preparations act by decreasing AI-2 levels, impairing *luxS* expression, or interfering with AI-2 detection.

Recently, Ramić et al.^25^ developed a highly sensitive whole-cell biosensor assay based on the *V. harveyi* MM30 reporter strain to quantify AI-2 production by *C. jejuni* in different growth media and food models. This assay demonstrated a 100-fold greater sensitivity compared to high-pressure liquid chromatography in combination with a fluorescence detection (HPLC-FLD) method. The *V. harveyi* MM30 reporter strain lacks the ability to produce its own AI-2 signaling molecules, yet it exhibits a strong bioluminescent response upon detecting AI-2 in *C. jejuni* SGM and food models. Additionally, unlike *C. jejuni*, *V. harveyi* MM30 produces hepatocyte growth factor activator inhibitor type 1, which can further amplify the bioluminescent response to AI-2 and enable the detection of even lower AI-2 concentrations in the tested media. This method was also independently validated by HPLC-FLD, which showed that AI-2 concentrations increase linearly with cell density and are significantly higher in food systems compared to defined growth media. This suggests that AI-2 in *C. jejuni* functions more as a metabolic by-product than as a conventional intercellular signaling molecule. This biosensor technique represents a breakthrough in the detection and quantification of AI-2 in complex matrices and could elucidate *C. jejuni* behavior in food production. To this end, the current study is a continuation of our previously published study.^25^

Here, we investigated the effects of subinhibitory concentrations of lavender preparations (essential oil (LAEO), ethanol extracts of flowers (LAEF), and ethanol extracts of wastes after essential oil distillation (LAEW)) and pure compounds (linalool and linalyl acetate) on the intercellular signaling of *C. jejuni* NCTC 11168. Our comprehensive analysis has elucidated the effects of natural compounds on *C. jejuni* intercellular signaling. These findings are essential for developing new strategies to combat bacterial infections.

## Results

### Minimal inhibitory concentrations of lavender preparations and pure compounds against *C. jejuni*

To determine the appropriate subinhibitory concentrations, we first obtained minimal inhibitory concentration (MIC) values for lavender preparations (LAEO, LAEF, and LAEW) and pure compounds (linalool and linalyl acetate). These MIC values (Table S3) were established in our previous study.^26^ The 1/4 MIC was selected as the highest subinhibitory concentration that did not affect the growth of either wild-type (wt) *C. jejuni* NCTC 11168 and *luxS*-mutant 11168Δ*luxS* (lacking AI-2 production) in comparison with untreated cultures. Therefore, we concluded that at lower concentrations, the observed effects of the preparations and pure compounds are likely mediated through other cellular mechanisms, as growth impairment was ruled out.

### Bioluminescent responses of *V. harveyi* MM30 to *C. jejuni* SGM

**Figure 1:**
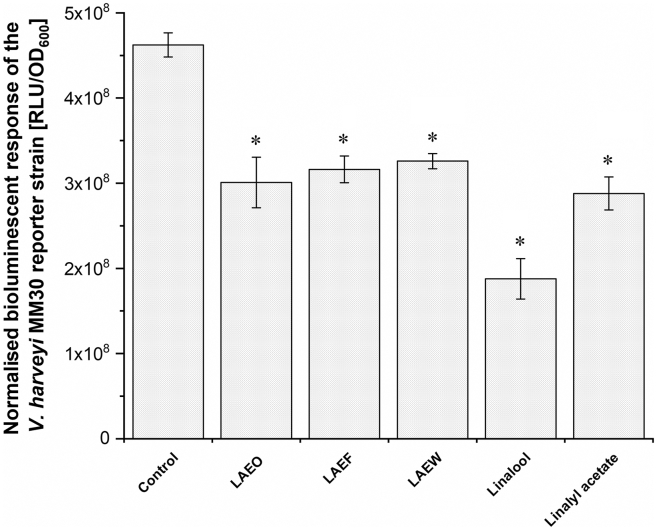
Bioluminescent responses of *V. harveyi* MM30 Spent growth media of wild-type *C. jejuni* cultured either without (control) or with subinhibitory concentrations (1/4 minimal inhibitory concentrations) of lavender preparations (lavender essential oil (LAEO), ethanol extracts of flowers (LAEF), and ethanol extracts of wastes after essential oil distillation (LAEW)) or pure compounds (linalool and linalyl acetate) were added to the reporter strain. The normalized bioluminescent response is displayed in relative luminescence units (RLUs) divided by the optical density [OD_600_] ± standard deviation ($\bar{x}$ (RLU/OD_600_) ± SD). ∗*p* < 0.05, parametric one-way ANOVA with Dunnett’s post hoc test.

The effects of LAEO, LAEF, LAEW, linalool, and linalyl acetate were assessed indirectly by measuring the bioluminescent response of *V. harveyi* MM30. Wt and *luxS*-mutant *C. jejuni* were incubated with or without LAEO, LAEF, LAEW, linalool, and linalyl acetate at subinhibitory concentrations (1/4 MIC). The SGM from these cultures was then added to the *V. harveyi* MM30. SGM from treated wt cultures significantly decreased the normalized bioluminescent response of V*. harveyi* MM30 compared to the SGM from untreated cultures (*p* < 0.05; Figure 1). The greatest decrease was observed with SGM from *C. jejuni* treated with linalool, whereas SGM from cultures treated with lavender preparations and linalyl acetate showed similar effects (Figure 1).

These findings suggest that lavender preparations and pure compounds decrease AI-2 concentrations either by influencing *luxS* gene expression, consequently LuxS protein abundance or by chemically inactivating AI-2 in the growth media. To further explore this, we directly quantified AI-2 concentrations in different SGMs.

### Quantification of AI-2 using HPLC-FLD

**Figure 2:**
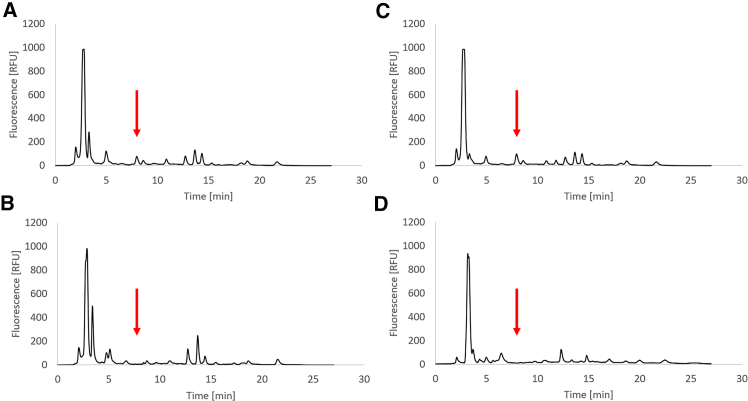
Chromatograms of spent growth media. *C. jejuni* was cultured in Mueller-Hinton broth. Data are represented as mean ± SD (A–D) (A) Wild-type control with its average peak area 15.46 ± 2. 84 [mV · min], (B) *luxS*-mutant control with its average peak area 6.67 ± 0.18 [mV · min], (C) wild-type cultures treated with lavender essential oil (1/4 minimal inhibitory concentration; average peak area 19.94 ± 5.25 [mV · min]; no statistically significant difference compared to untreated wild type; *p* = 0.36), and (D) *luxS*-mutant cultures treated with lavender essential oil (1/4 minimal inhibitory concentration; average peak area 6.82 ± 0.14 [mV · min]; no statistically significant difference compared to untreated luxS-mutant *p* = 0.57). Red arrows indicate the retention time of AI-2 (7.9 min, as described in Ramić et al. (2022b)) in wild-type cultures (A, C) or the absence of AI-2 in *luxS*-mutant cultures (B, D).

The concentrations of AI-2 in the SGM of wt *C. jejuni* cultures that were untreated (Figure 2A) or treated with LAEO (Figure 2C), LAEF, LAEW, linalool, and linalyl acetate (at 1/4 MIC) were quantified using HPLC-FLD (Figure 2). SGM of the *luxS*-mutant, which does not produce AI-2,^12^ cultured under identical conditions as wt, was used as the negative control (Figures 2B and 2D).

**Figure 3:**
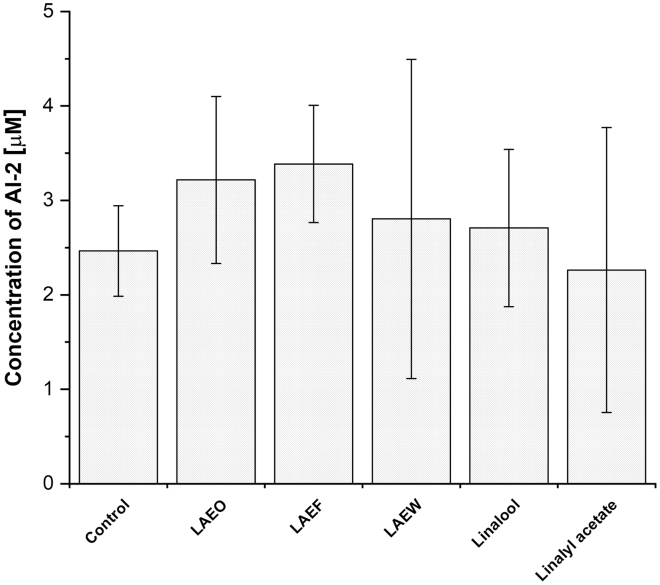
The concentrations of autoinducer-2 (AI-2) in the spent growth media of wild-type *C. jejuni* Bacteria were cultured without (control) or with lavender essential oil (LAEO), ethanol extracts of flowers (LAEF), ethanol extracts of wastes after essential oil distillation (LAEW), linalool, or linalyl acetate at the subinhibitory concentrations (1/4 minimal inhibitory concentration) determined by high-pressure liquid chromatography with a fluorescence detector. The results are presented as mean ± standard deviation ($\bar{x}$ (μM) ± SD).

Analysis of the retention time peak areas corresponding to AI-2 in all wt SGMs revealed no significant differences in AI-2 concentrations between the treated and untreated SGM (*p* > 0.05; Figure 3).

### Effects of LAEO, linalool, and linalyl acetate on *luxS* expression

The expression of *luxS* in wt *C. jejuni* was determined using real-time PCR (qPCR). Normalized *luxS* expression levels in wt cultures treated with LAEO, linalool, or linalyl acetate did not differ significantly from the untreated control (*p* > 0.05; Figure S1), which consisted of wt cultures treated only with the solvent used in treated samples (1% DMSO).

### Effects of linalool and linalyl acetate on the protein LuxS abundance

The relative abundance of the LuxS protein was evaluated using a label-free quantification approach and calculated using normalized spectral abundance factor method (NSAF). Log_2_-transformed NSAF values from wt cultures treated with linalool or linalyl acetate were compared to those from the solvent control (1% DMSO). No statistically significant differences in LuxS protein abundance were observed between the treated and control groups (*p* > 0.05; Table S4), indicating that neither linalool nor linalyl acetate had a measurable impact on LuxS levels under the tested conditions.

Using three independent methods, we confirmed that the lavender preparations and pure compounds neither affected the *luxS* gene expression, LuxS protein abundance, nor directly decreased AI-2 concentrations in the SGM of treated wt cultures. Based on these findings, we hypothesized that active compounds remaining in the SGM may interfere with the *V. harveyi* MM30 biosensor by disrupting the detection of available AI-2 molecules.

### The effects of different wt SGM on the bioluminescent response of *V. harveyi* MM30

**Figure 4:**
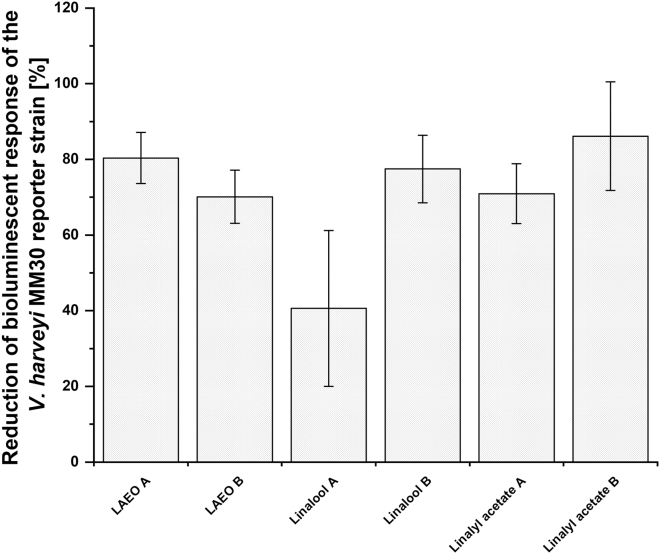
The relative decreases in the bioluminescent responses of *V. harveyi* MM30 to two different spent growth media (SGM) types The first SGM type (A) was prepared from wild-type cultures treated with 1/4 of the minimal inhibitory concentrations of lavender essential oil (LAEO), linalool, and linalyl acetate for 24 h. The second SGM type (B) was prepared from untreated wild-type cultures and was supplemented with 1/4 of the minimal inhibitory concentrations of LAEO, linalool, and linalyl acetate after the 24 h incubation. The data are presented as mean ± standard deviation ($\bar{x}$ (%) ± SD).

To determine whether the observed decreased bioluminescent response of *V. harveyi* MM30 to *C. jejuni* SGM is due to direct interactions between lavender preparations and AI-2/*luxS* or indirect effects via the *V. harveyi* MM30 AI-2 receptor, two types of SGM were prepared. The first was prepared from wt cultures treated with 1/4 MIC of LAEO, linalool, and linalyl acetate for 24 h. The second was prepared from untreated wt cultures cultivated for 24 h, after which 1/4 MIC subinhibitory concentrations of LAEO, linalool, and linalyl acetate were added to the SGM. These SGM were added to *V. harveyi* MM30, and bioluminescence was measured. This approach ensures a comprehensive understanding of the impact of LAEO, linalool, and linalyl acetate on bacterial communication. The relative decreases in the bioluminescent responses of *V. harveyi* MM30 did not significantly differ between the two SGM types (*p* > 0.05; Figure 4). This indicates that the decreased bioluminescent response could be the effect of pure compounds on the selected biosensor strain but not on *luxS*-related pathways in *C. jejuni.*

### *In silico* analysis of LuxP interactions with tested compounds via CB-Dock2

To support our hypothesis that active compounds from lavender preparations may interfere with the AI-2 receptor in the biosensor strain *V. harveyi* MM30, we performed *in silico* docking simulations using the LuxP receptor protein and two major lavender-derived compounds: linalool and linalyl acetate. These were compared against AI-2 (the native signaling molecule) and DMSO (the solvent, used as a negative control).

**Figure 5:**
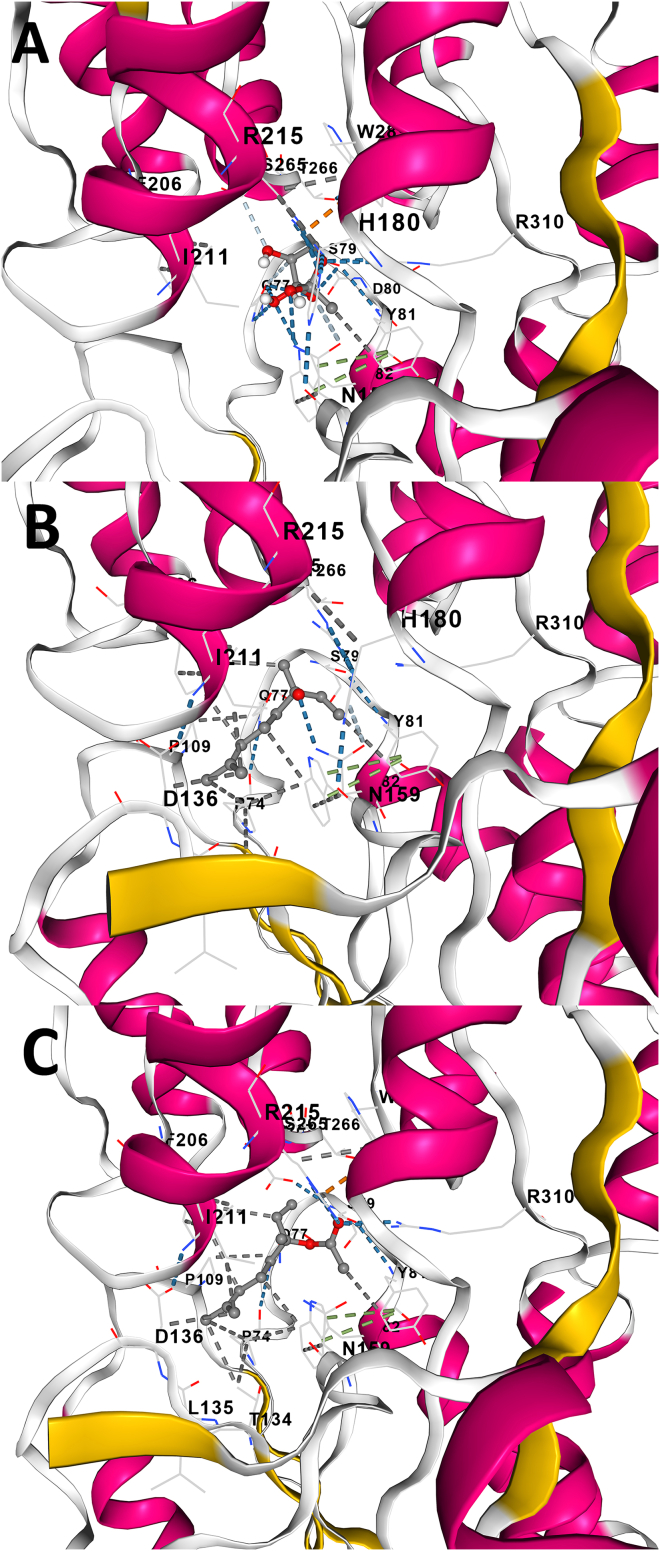
Molecular docking of AI-2 and lavender compounds to LuxP (A–C) Predicted docking poses of autoinducer-2 (A), linalool (B), and linalyl acetate (C) within the LuxP receptor binding site, modeled using AutoDock Vina. Key interacting protein amino acid residues are indicated by their one-letter codes and position numbers in the protein sequence. Ligands are shown in stick representation; key residues are labeled and colored by atom type. Docking models were generated using CB-Dock2.

The CB-Dock2 program identified a medium-sized binding cavity in the LuxP protein with a volume of 905 Å^3^ and center coordinates at (x = −16, y = −13, z = 9). This cavity was used for all docking simulations (Figure 5).

Binding affinities to this cavity were evaluated using AutoDock Vina, which provides Vina scores—a more negative value indicates stronger predicted binding as well as amino acid contact residues (Table S5).

The reference compound AI-2 exhibited the strongest predicted binding (−6.9 kcal/mol). Linalyl acetate showed a very similar binding affinity (−6.7 kcal/mol), suggesting that it could compete with AI-2 for the same binding site. Linalool also demonstrated notable binding potential (−6.3 kcal/mol), although slightly weaker. By contrast, the solvent DMSO showed poor binding (−3.1 kcal/mol), as expected for a compound with no specific interaction.

## Discussion

Preventing campylobacteriosis requires combating *Campylobacter* in food-processing environments through stricter hygiene practices, especially as infections in humans can occur at very low bacterial doses. The number of unreported cases is likely much higher than the number of known cases,^1^ and the prevalence of *C. jejuni* strains resistant to antibiotics and biocides, mainly isolated from poultry, is high and still increasing.^27^^,^^28^^,^^29^ An even greater challenge is posed by biofilm-associated infections, in which microbes have significantly higher antibiotic resistance compared to planktonic forms, leading to persistent contamination and recurrent infections.^30^ To this end, new approaches for combating bacterial infections are required, and the use of natural antimicrobials at subinhibitory concentrations is a promising method to avoid increasing antimicrobial resistance. Focusing on bacterial biofilms and interspecies signaling is emerging as an innovative approach for intervention strategies and offers insights for the prevention and treatment of *C. jejuni* infections without exacerbating the problem of antibiotic resistance.^31^

Lavender preparations exert anti-bacterial and anti-biofilm activity by affecting the initial steps of biofilm formation (e.g., adhesion and motility), as well as the downregulation of transmembrane proteins, many involved in the iron uptake systems and stress response in *C. jejuni*.^26^ Additionally, Ramić et al.^24^ confirmed that lavandin (*Lavandula x intermedia*) preparations at subinhibitory concentrations exert anti-bacterial effects on *C. jejuni* by affecting intercellular signaling, adhesion, and biofilm formation. This suggests that lavender and lavandin can be used as antimicrobial agents to control *C. jejuni* biofilm development.

In Ramić et al.,^25^ we investigated the effects of lavender preparations on the intercellular communication of *C. jejuni* via an indirect approach, i.e., by measuring the bioluminescent response of different *V. harveyi* reporter strains to *C. jejuni* SGM. *V. harveyi* MM30 was selected as the optimal strain to study the intercellular communication of *C. jejuni* because of its robust and sensitive response to AI-2 produced by *C. jejuni*.^25^ The versatility of the method has been confirmed in Gram-positive *Staphylococcus aureus*.^32^

Our results reveal decreased bioluminescent responses of *V. harveyi* MM30 when exposed to SGM of *C. jejuni* treated with lavender preparations and pure compounds, compared to SGM of untreated *C. jejuni* (Figure 1). Similar results were reported for ethanolic extracts of the plants *Euodia ruticarpa* and *Rhodiola rosea* and the essential oil of *Lavandula hybrida*, all of which decreased the bioluminescent response of *V. harveyi* MM30, after the addition of SGM of treated *C. jejuni*.^19^^,^^22^^,^^33^ However, it remained unclear whether the plant preparations affect AI-2 concentration, *luxS* expression, LuxP abundance, or even the AI-2 receptor.

Our findings indicate that the tested lavender preparations and pure compounds do not reduce AI-2 concentrations in *C. jejuni* SGM. Together with unchanged *luxS* expression and LuxS protein abundance, these data support that lavender-derived compounds do not interfere with AI-2 production in *C. jejuni*.

Furthermore, our results revealed that LAEO, linalool, and linalyl acetate do not significantly alter *luxS* expression in *C. jejuni* (Figure S1), or LuxS abundance (Table S4), which may explain the unchanged AI-2 concentration in the SGM. Similarly, Wagle et al.^34^ found that the pure compound eugenol does not affect *luxS* expression in *C. jejuni*, whereas *trans*-cinnamaldehyde and carvacrol even increased *luxS* expression. Our previous research^26^ revealed that LAEO (at subinhibitory concentrations) affects the expression of numerous genes in *C. jejuni*. Of the 326 differentially expressed genes, 188 were downregulated, including genes critical for motility, flagellar synthesis, biofilm formation, and outer membrane protein production, all of which are crucial for successful colonization and pathogenicity. Furthermore, the expression of the CmeABC efflux pump system was increased, indicating a significant bacterial stress response.

Moreover, luxS expression remained unaffected, corroborating the results of this study. Accordingly, our data indicate that the selected plant preparations do not alter AI-2 concentration or luxS transcription in C. jejuni. This observation is in agreement with our previous studies demonstrating a linear correlation between AI-2 levels and *C. jejuni* cell density,^24^ as well as the predominant involvement of luxS in the central metabolism of this organism.^18^ Altogether, these findings are consistent with previous evidence suggesting that AI-2 in *C. jejuni* is more closely associated with metabolic activity than with canonical quorum-sensing signaling.^18^^,^^24^

Nevertheless, the decreased bioluminescence in *V. harveyi* MM30 after the addition of SGM from treated *C. jejuni* remained unresolved. Thus, we investigated whether lavender-derived compounds may interfere with AI-2 detection by the *V. harveyi* biosensor. Although effects on other QS systems cannot be entirely excluded, the experimental design allowed us to primarily attribute the observed response to the modulation of AI-2-dependent signaling.

Comparable bioluminescent responses of *V. harveyi* MM30 to both SGM types suggest that the observed signal reduction is not mediated via altered AI-2 production by *C. jejuni*. This suggests that lavender preparations and pure compounds interfere with AI-2 detection in the *V. harveyi* MM30 biosensor, potentially through interactions with the LuxP receptor, leading to a reduced bioluminescent response.

To further substantiate our hypothesis that lavender-derived compounds can interfere with bacterial quorum sensing, we conducted *in silico* docking simulations comparing linalool and linalyl acetate to the native signaling molecule AI-2. Using the CB-Dock2 platform, we identified a medium-sized binding cavity within the LuxP receptor of *V. harveyi*. As anticipated, AI-2 exhibited the strongest predicted binding affinity. Linalyl acetate demonstrated a comparable binding affinity, suggesting potential competition with AI-2 at the LuxP binding site. Linalool also showed significant binding potential, albeit slightly weaker (Table S5).

Although the greatest decrease in bioluminescence was observed with linalool (Figure 1), this compound exhibited a slightly lower predicted binding affinity to the LuxP receptor compared to linalyl acetate in the *in-silico* docking simulations. This apparent discrepancy may be explained by considering the higher MIC values of linalool. It is likely that a greater concentration of linalool remained active in the system. This higher molecular abundance may have enabled more linalool molecules to compete effectively for binding at the active site of the LuxP receptor, despite its slightly weaker individual binding affinity. The docking analysis should be interpreted as predictive and does not constitute direct experimental evidence of LuxP inhibition.

These findings align with previous studies indicating that natural compounds can modulate quorum-sensing pathways. For instance, Shekarappa et al.^35^ utilized *in silico* screening to identify potential AI-2 inhibitors, highlighting the efficacy of computational approaches in discovering quorum-sensing modulators. Additionally, research on Romanian lavender essential oils demonstrated that components such as linalool and linalyl acetate could interact with bacterial surface proteins, supporting their role in disrupting microbial adhesion and consequently biofilm.^36^ Moreover, this indicates that plant preparations can alter interspecies communication, which can directly or indirectly affect bacterial properties such as biofilm formation and pathogenicity. Such a strategy could help us control interspecies biofilms, addressing one of the world’s largest problems regarding food safety and public health.

### Conclusions

SGM of *C. jejuni* treated with lavender preparations (LAEO, LAEF, and LAEW) and pure compounds (linalool and linalyl acetate) at subinhibitory concentrations effectively decreased the bioluminescent response of *V. harveyi* MM30. However, these preparations and pure compounds did not decrease AI-2 concentration, *luxS* expression, nor LuxS abundance in *C. jejuni*. The lavender preparations and pure compounds were predicted by in silico analyses to interact with *V. harveyi* AI-2 receptor. However, direct experimental validation, for example using biophysical approaches such as isothermal titration calorimetry, will be required to confirm these interactions. Nevertheless, the observed binding poses suggest that these compounds may interfere with AI-2 detection by mimicking structural features of the AI-2 binding site, thereby representing a potential strategy for modulating interspecies communication and AI-2-dependent biofilm formation. These indirect mechanisms should be further explored to elucidate the use of natural antimicrobials for combating bacterial infections by disrupting the bacterial coordinated response, such as biofilm formation, virulence factor expression, and motility changes. Overall, this study emphasizes the importance of understanding microbial communication pathways for effective food control and disease prevention, demonstrating that pure compounds from lavender can directly compete with signaling molecules and disrupt intercellular communication.

### Limitations of the study

This study has limitations that should be acknowledged. First, the interaction between lavender-derived compounds and the AI-2 detection system in *Vibrio harveyi* was inferred from functional biosensor assays and supported by in silico docking analyses. While these computational results are predictive and provide mechanistic insight, direct experimental validation of ligand-receptor interactions was not performed.

Second, the study focused specifically on AI-2–dependent signaling using the *V. harveyi* MM30 biosensor. Potential effects of lavender-derived compounds on other quorum-sensing systems, such as CAI-1, HAI-1, or nitric oxide-based signaling pathways, were not directly assessed and therefore cannot be excluded.

## Resource availability

### Lead contact

Requests for further information and resources should be directed to and will be fulfilled by the lead contact, Anja Klančnik (anja.klancnik@bf.uni.lj.si).

### Materials availability

The lavender preparations used in this study, including essential oil and ethanol extracts of *Lavandula angustifolia*, are the same as those previously described and fully characterized by Ramić et al. (2021).

### Data and code availability

• Sequencing data have been deposited in the NCBI Sequence Read Archive (SRA) under BioProject accession number PRJNA747749, with individual SRA accession numbers: SRR15183210, SRR15183211, SRR15183212, SRR15183213, SRR15183214, SRR15183215, SRR15183216, SRR15183226, and SRR15183227. Mass spectrometry proteomics data are available via the ProteomeXchange Consortium through the PRIDE partner repository under the dataset identifier PXD067187. All deposited data are publicly available as of the date of publication.
• This paper does not report original code.
• The data supporting the findings of this study are available from the lead author upon request.

## Acknowledgments

We thank Dr. Eva Lasic for editing and reviewing a draft of this manuscript, and Manca Volk for her valuable assistance with qPCR analyses. This research was funded by the Slovenian Research and Innovation Agency (grant no. J4-2542, J4-3088, J4-4548, and P4-0116).

## Author contributions

Conceptualization, D. J., S.S.M., and A.K.; formal analysis, B.J. and D.J.; investigation, B.J. and D.J.; methodology, B.J., D.J., and A.K.; resources, D. J., S.S.M., and A.K.; validation, B.J. and D.J.; visualization, B.J., D.J., and A.K.; writing – original draft preparation, B.J. and D.J.; funding acquisition, S.S.M. and A.K.; project administration, S.S.M. and A.K.; supervision, S.S.M. and A.K.; writing – review and editing; B.J., D.J., S.S.M., and A.K.

## Declaration of interests

The authors declare no competing interests.

## STAR★Methods

### Key resources table

**Table:** 

REAGENT or RESOURCE	SOURCE	IDENTIFIER
**Bacterial and virus strains**		

*Campylobacter jejuni* NCTC 11168	National Collection of Type Culture	NCTC 11168
*Campylobacter jejuni* NCTC 11168Δ*luxS*	Our laboratory	NCTC 11168Δ*luxS*
*Vibrio harveyi* MM30	prof. dr. Tom Defoirdt, from Center for Microbial Ecology and Technology, Ghent University	MM30

**Chemicals, peptides, and recombinant proteins**		

Lavender preparations	Defined in Ramić et al.^26^	NA
Linalool	Sigma-Aldrich, USA	CAT#L2602
Linalyl acetate	Sigma-Aldrich, USA	CAT#W263613
(S)-4,5-dihydroxy-2,3-pentanedione	Carbosynth, UK	CAT#MD170303

**Critical commercial assays**		

PureLink RNA mini kit	Thermo Fisher Scientific, USA	CAT#12183018A
PureLink DNase kit	Thermo Fisher Scientific, USA	CAT#12185010
Qubit RNA HS kit	Thermo Fisher Scientific, USA	CAT#Q32852
BacTiter-Glo reagent	Promega, USA	CAT# G8230

**Deposited data**		

RNA sequencing data - NCBI, BioProject: PRJNA747749 (SRR15183210, SRR15183211, SRR15183212, SRR15183213, SRR15183214, SRR15183215, SRR15183216, SRR15183226, SRR15183227)	This paper and Ramić et al.^18^	https://www.ncbi.nlm.nih.gov/bioproject/PRJNA747749
Mass spectrometry proteomics; PRIDE: PXD067187	This paper	https://www.ebi.ac.uk/pride/archive/projects/PXD067187

**Oligonucleotides**		

Probes (*ilvC*, *rpoA*, *luxS*)	TaqMAN, Thermo Fisher Scientific, USA	CAT#4316034
Gene primers (*ilvC*, *rpoA*, *luxS*)	Promega, USA	NA

**Software and algorithms**		

Reffinder program	Xie et al.^37^	https://www.ciidirsinaloa.com.mx/RefFinder-master/
Proteome Discoverer (v1.4.0.288)	Thermo Fisher Scientific, USA	https://www.thermofisher.com/si/en/home/industrial/mass-spectrometry/liquid-chromatography-mass-spectrometry-lc-ms/lc-ms-software/multi-omics-data-analysis/proteome-discoverer-software.html
IBM SPSS Statistics 23	Statsoft Inc., USA	https://www.ibm.com/us-en
OriginPro	OriginLab, Northampton, USA	https://www.originlab.com/

### Experimental model and study participant details

#### Bacterial strains and growth conditions

Wild-type (wt) *C. jejuni* NCTC 11168 and *luxS*-mutant 11168Δ*luxS* (lacking AI-2 production^12^) strains were maintained in a solution of Mueller-Hinton (MH) broth (Oxoid, UK) and glycerol (Kemika, Croatia) at −80 °C, as described by Ramić et al.^26^ and Plummer et al.^38^ Bacteria were plated onto Karmali agar (Oxoid, UK) with selective additives and incubated under microaerobic conditions (5% O_2_, 10% CO_2_, 85% N_2_) at 42 °C for 48 h. Wt cultures were further cultivated on MH agar (Oxoid, UK) without antibiotics, whereas *luxS*-mutant cultures were cultivated on MH agar supplemented with kanamycin (30 mg/L) (Sigma Aldrich, USA). The cultures were incubated under microaerobic conditions at 42 °C for 24 h. Inoculum was prepared in MH broth and adjusted to an OD_600_ of 0.1 a.u. Colony counts for both strains were performed under the same growth conditions as previously described, and the results were expressed as colony-forming units (CFU)/mL.

*Vibrio harveyi* MM30 was used for the biosensor assay^39^ under the conditions described by Ramić et al.^25^ and stored at −80 °C in medium consisting of 20% glycerol and 80% autoinducer bioassay growth medium containing 0.02 g/L NaCl, 0.01 g/L MgSO_4_ × 7 H_2_O, 0.002 g/L casamino acid, 1 M phosphate-buffered saline, 0.1 M L-arginine, and 50% (v/v) glycerol.^25^^,^^40^ The strain was cultured aerobically at 30 °C for 16 h in autoinducer bioassay growth medium prior to the experiments.

### Method details

#### Lavender preparations and key compounds

This study included the lavender (*Lavandula angustifolia*) preparations LAEO, LAEF, and LAEW and the key pure compounds of lavender essential oil—linalool and linalyl acetate.^26^ The production processes and chemical characterization of LAEO, LAEF, and LAEW were described in detail previously.^26^ LAEO was produced by hydrodistillation of dried flowers, whereas LAEF and LAEW were obtained from the Soxhlet extraction from flowers or waste material after distillation, respectively. Key pure compounds in LAEO were identified and quantified using gas chromatography-mass spectrometry.

#### Minimal inhibitory concentration (MIC) determination

The MIC values of lavender preparations (LAEO, LAEF, and LAEW) and pure compounds (linalool and linalyl acetate) against wt and *luxS*-mutant *C. jejuni* were obtained using the broth microdilution method, as described by Klančnik et al.^41^ In short bacterial cultures prepared as described above were added to 2-fold serially diluted preparations or pure compounds in MH broth in Nunc 96-well polystyrene microtiter plates (Thermo Fisher, USA). After 24 h of incubation, the MIC value was determined as the lowest concentration of a tested preparation or compound where no metabolic activity was observed using BacTiter-Glo reagent (Promega, USA) measured with a microplate reader (Varioskan LUX, Thermo Fisher, USA).

#### Biosensor assay with *V. harveyi* MM30

The bioluminescent response of the *V. harveyi* reporter strain is often used as an indirect method to study bacterial intercellular communication.^42^ Our study assessed the effects of LAEO, LAEF, LAEW, linalool, and linalyl acetate on the intercellular communication of wt *C. jejuni* cultures. We measured the bioluminescent response of *V. harveyi* MM30 using a microplate reader (Varioskan LUX, Thermo Scientific, Waltham, MA, USA) following the method described by Ramić et al.^25^ Briefly, *V. harveyi* MM30 culture were diluted to approximately 10^3^ CFU/mL. The SGM of *C. jejuni* cultures was prepared in two different ways and then added to *V. harveyi* MM30. First, wt and *luxS*-mutant *C. jejuni* (lacking AI-2 production^12^) were cultivated for 24 h with or without lavender preparations and pure compounds at subinhibitory concentrations (1/4 MIC) which were previously confirmed not to affect bacterial growth using the CFU method.^26^ Following incubation, cultures were filtered through 0.22 μm filters (Sartorius, Germany) to acquire SGM. Second, wt and *luxS*-mutant *C. jejuni* were cultivated for 24 h without plant preparations, after which SGM were acquired and then supplemented with LAEO and pure compounds at the same subinhibitory concentrations. SGM were added to *V. harveyi* MM30 cultures at a final concentration of 5% (v/v) in 96-well microtiter plates. Blank samples and controls were prepared with wt SGM, *luxS*-mutant SGM, or MH broth. Luminescence and growth (OD_600_) were measured at 30 min intervals for 15 h at 30 °C. Bioluminescent responses of *V. harveyi* MM30 were measured in relative luminescence units. Appropriate blank values were subtracted from the measured bioluminescent response for each sample and normalised to the measured OD_600_ value. The decrease in bioluminescence was calculated using the following equation:

$$\text{Bioluminescence}\;\text{reduction}\;\left(\%\right)=\frac{Treated\;wt-Treated\;luxS\;mutant}{Untreated\;wt-Untreated\;luxS\;mutant}\times 100\%$$

Treated wt refers to the normalised bioluminescent response of *V. harveyi* MM30 after the addition of treated or subsequently treated wt SGM.

Treated *luxS*-mutant refers to the normalised bioluminescent response of *V. harveyi* MM30 after the addition of treated or subsequently treated *luxS*-mutant SGM.

Untreated wt refers to the normalised bioluminescent response of *V. harveyi* MM30 after the addition of untreated wt SGM.

Untreated *luxS*-mutant refers to the normalised bioluminescent response of *V. harveyi* MM30 after the addition of untreated *luxS*-mutant SGM.

All of the experiments for acquisition of treated or untreated *C. jejuni* wt or *luxS*-mutant SGM were carried out as three biological independent experiments, with each biological experiment consisting of three or more technical replicates.

#### Quantification of AI-2 with HPLC-FLD

AI-2 in wt *C. jejuni* cultures (untreated and treated with LAEO, LAEF, LAEW, linalool, and linalyl acetate) was quantified using HPLC-FLD methods described in detail by Ramić et al.^25^ *LuxS*-mutant *C. jejuni* was used as a negative control, as it does not produce AI-2. A calibration curve was obtained using the AI-2 precursor (S)-4,5-dihydroxy-2,3-pentanedione (Carbosynth, UK). For AI-2 detection, samples were derivatized with 0.2 mg/mL diaminonaphthalene (Sigma Aldrich, Germany) at 90 °C for 40 min, generating a fluorescent derivative with specific excitation and emission wavelengths (271 and 503 nm, respectively). Samples were analyzed by HPLC-FLD with a C-18 reversed-phase column at 30°C.^25^

#### Determining the effects of LAEO, linalool, and linalyl acetate on *luxS* gene expression using qPCR

The expression of the *luxS* gene in planktonic wt *C. jejuni* cultures was determined using the ViiA 7 real-time PCR (qPCR) system (Thermo Fisher Scientific, Applied Biosystems, CA, USA). Wt *C. jejuni* were cultured for 16 h in MH broth (Oxoid, UK) at 42 °C under microaerobic conditions (5% O_2_, 10% CO_2_, and 85% N_2_). After 16 h, LAEO, linalool, or linalyl acetate were added at subinhibitory concentrations (1/4 MIC) to the cultures, which were incubated for another 30 min under the same conditions. Stock solutions of the preparations were prepared in 1% dimethyl sulfoxide (DMSO; Merck, Germany), and thus the negative control (wt culture) was incubated for 30 min with 1% DMSO, which does not affect *C. jejuni* growth.^18^ Afterward, cells were centrifuged at 5000 × g for 5 min at 4 °C and resuspended in 1 mL of MH broth to a cell concentration of 10^9^ CFU/mL.

Isolation and purification of total RNA were performed using TRIzol reagent (Sigma Aldrich, USA) and the PureLink RNA mini kit (Thermo Fisher Scientific, USA). The DNase enzyme from the PureLink DNase kit (Thermo Fisher Scientific, USA) was used for the purification of isolated RNA from genomic DNA. RNA quantification was performed by fluorometry on the Qubit 4 fluorometer with the Qubit RNA HS kit (both from Thermo Fisher Scientific, USA). mRNA from total RNA was isolated using NEXTflex PolyA magnetic beads (PerkinElmer, USA).

For qPCR, unique primer oligonucleotides and a FAM-MGB probe (TaqMAN, Thermo Fisher Scientific, USA) (Table S1) were designed using the BLAST program (National Center for Biotechnology Information, USA). Reference genes for data normalisation were selected based on the RNA sequencing results described by Ramić et al.^26^ The genes *ilvC* and *rpoA*, which did not show changes in differential gene expression levels, were selected.

The TaqMAM Universal Master Mix II with uracil-N-glycosylase (Thermo Fisher Scientific, USA) was used to prepare the reaction mixture for qPCR, according to the manufacturer’s instructions. The amplification conditions used for qPCR are provided in Table S2.

The amplification efficiency was calculated based on the calibration curve, for which dilution series for all samples were obtained. After analysing the data obtained by qPCR, the suitability of the reference genes was verified using the Reffinder program.^37^ The Pfaffl method was used to determine changes in gene expression.^43^ Data normalization was performed as described by Vandesompele et al.^44^

#### Determining the effects of linalool and linalyl acetate on LuxS protein abundance using quantitative proteomics

Biomass required for the proteomic analysis of treated and untreated wt *C. jejuni* was obtained in the same manner as described for qPCR analysis. Protein isolation, purification and quantitative proteomics procedures were performed as previously described by Ramić et al.^18^ Briefly, bacterial biomass was lysed in lysis buffer using rapid freeze thaw cycles, followed by homogenisation using zirconia glass beads (Carl Roth, Germany) in a Bullet Blender (Next Advance, USA). Protein concentration was determined using the Bradford assay. Crude protein extracts were purified using Nanosep 30K Omega filters microcentrifuges (Pall, USA) according to the protocol by Distler et al.^45^ Digested peptides were separated and analyzed by nano liquid chromatography (Thermo Scientific, Ultimate RSLC 3000) coupled in line with a Q Exactive mass spectrometer equipped with an Easy-Spray ion source (Thermo Fischer Scientific, USA). Tandem mass spectra were analyzed using the SEQUEST HT algorithm, part of Proteome Discoverer software (v1.4.0.288; Thermo Fisher Scientific). Spectra were searched against a custom database comprising 1,632 protein sequences, including those from *C. jejuni* NCTC 11168 (UniProt release: September 16, 2021) and common contaminants. Protein identification was filtered using a false discovery rate threshold of <1%. Quantitative analysis was based on a label-free quantification approach, with relative protein abundance estimated using the Normalized Spectral Abundance Factor method (NSAF).^46^

#### *In silico* docking of selected compounds on LuxP receptor

To evaluate potential binding of the tested pure compounds (linalool and linalyl acetate) within the AI-2 binding site of the *V. harveyi* LuxP receptor, we employed CB-Dock2, a web-based, cavity-guided, protein–ligand docking tool. CB-Dock2 automatically detects ligand-binding pockets using the CurPocket algorithm, estimates the cavity center and dimensions, and adjusts the docking box to the shape and size of the input ligands. The docking calculations were conducted using AutoDock Vina, which predicts binding poses and affinities based on a scoring function. The crystal structure of LuxP protein (PDB: 1JX6), originally described by Chen et al.,^47^ was used as the receptor model. The ligands linalool with SMILES specification C=C[C@](C)(O)CCC = C(C)C and linalyl acetate with SMILES specification C=C[C@@](C)(CCC = C(C)C)OC(C) = O were tested against AI-2 with SMILES specification[B-]1(O[C@@]2([C@](O1)([C@H](CO2)O)O)C)(O)O, whereas DMSO with SMILES specification CS(=O)C was used as a negative control. All were uploaded in SDF format, whereas the protein was uploaded in PDB format. The FitDock method was employed to perform the docking within the predicted cavities. Protein–ligand complexes were visualized and exported directly from the CB-Dock2 platform for graphical representation.^48^

### Quantification and statistical analysis

The experiments were conducted in three biological replicates, with each set comprising three or more technical replicates (*n* = 9). Data are presented as mean ± standard deviations ($\bar{x}$ ± SD). Statistical analyses were conducted using IBM SPSS Statistics 23 (Statsoft Inc., USA). Prior to analysis, the normality of the data distribution and the homogeneity of variances were assessed using the Kolmogorov-Smirnov test and test for homogeneity of variances, respectively. Significance levels were determined using parametric one-way ANOVA with Dunnett’s post hoc test with results considered significant if *p* < 0.05. More detailed descriptions are listed in the figure legends and results section. Figures were created using OriginPro (OriginLab, Northampton, USA).
