# Supplementary material for: Lavender compounds interfere with AI-2 dependent bioluminescence in Vibrio harveyi without affecting LuxS signaling in Campylobacter jejuni
Source: iScience. 2026 Mar 7;29(4):115283. doi: 10.1016/j.isci.2026.115283 (PMC13049666; doi:10.1016/j.isci.2026.115283)
Supplement: Document S1. Figure S1 and Tables S1–S5 [file mmc1.pdf]

iScience, Volume *29*

Supplemental information

Lavender compounds interfere with AI-2 dependent bioluminescence in *Vibrio harveyi* without affecting LuxS signaling in *Campylobacter jejuni*

Blaž Jug, Dina Jug, Sonja Smole Možina, and Anja Klančnik

Supplementary Table S1. The primers used for rtPCR analysis of *luxS* gene expression.

Gene	Forward oligonucleotide primer	Reverse oligonucleotide primer	Probe
ilvC	TGCAGAATACGGCGATTACATCA	CATCGCTTTTTTAGTCTCT TCAGTG	CAGGGCCAAAG ATTAT
rpoA	GTCTTGAAAAAGCAGGAGTGGTTT	AAGCCCTGCAAGTTCACTT ACA	CTCATCAAAGC AAGCTC
luxS	AGGATTTATGAGAGATCATCTTAAT TCAAATTCAGTT	CAATACTTTTCTCATCAGG TGTTCCAAT	ACCCGTGCGAC AACC

Supplementary table S2. Amplification conditions for rtPCR.

Step	Temperature [°C]	Time [min:s]	Number of cycles
Uracil-N-glycosylase incubation	50	2:0	1
Enzyme activation	95	10:0	1
Denaturation	95	0:15	40
Annealing	60	1:0	

Supplementary table S3. Minimal inhibitory concentrations (MIC) and subinhibitory concentrations (1/4 MIC) of the lavender preparations and pure compounds used to treat wild-type and *luxS*-mutant *C. jejuni* (Ramić et al., 2021). LAEO: lavender essential oil; LAEF: ethanol extracts of flowers; LAEW: ethanol extracts of wastes after essential oil distillation.

Compounds	MIC (mg/L)	1/4 MIC (mg/L)
Lavender essential oil	0.25 ± 0.06	0.0625
Ethanol extracts of flowers	1.00 ± 0.25	0.25
Ethanol extracts of wastes after essential oil distillation	1.00 ± 0.25	0.25
Linalool	0.25 ± 0.06	0.0625
Linalyl acetate	0.20 ± 0.05	0.05

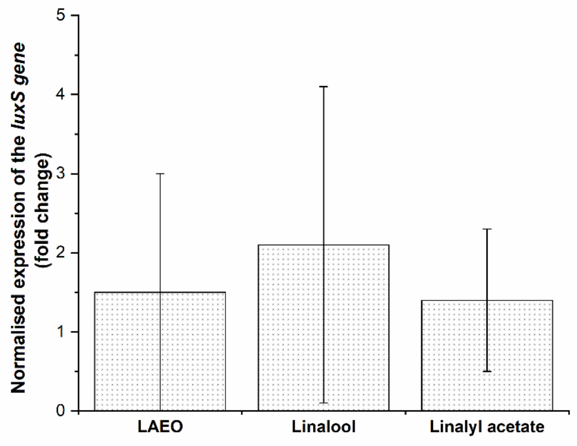

Supplementary figure 1. Normalised *luxS* expression in wild-type *C. jejuni* after treatment with lavender essential oil (LAEO), linalool, and linalyl acetate at subinhibitory concentrations (1/4 minimal inhibitory concentration). The changes in gene expression were determined using the Pfaffl method (Pfaffl, 2001), with data normalisation as described by Vandesompele et al. (2002). Data are presented as mean ± standard deviation.

Supplementary table S4. Relative abundance of the LuxS protein in wt *C. jejuni* cultures treated with linalool or linalyl acetate. Log_2-transformed NSAF values were compared to those from the solvent control (1% DMSO). Fold changes and corresponding p-values were calculated to assess differences in protein abundance between treated and control groups.

Treated wt *C. jejuni* with:	Log_2-transformed NSAF value ± SD	Log_2 fold change	p-value
solvent control (DMSO)	-2.65 ± 0.23	/	/
linalool	-3.05 ±0.32	0.40	0.91
linalyl acetate	-3.25 ±0.09	0.60	0,14

Supplementary table S5. Vina scores of tested compounds docked to the AI-2 binding site on the LuxP protein.

Ligand	Vina score (kcal/mol)	Amino acid contact residues:
AI-2	−6.9	PRO74 GLN77 SER79 ASP80 TYR81 TRP82 THR107 ARG108 PRO109 ASN110 ALA111 ILE113 GLN116 THR134 LEU135 ASP136 THR137 ARG139 HIS140 ASN159 HIS180 PHE206 SER207 GLU208 ILE211 ARG215 CYS264 SER265 THR266 ASP267 TRP289 GLY290 GLU295 ARG310
Linalool	−6.3	PRO74 GLN77 SER79 TYR81 TRP82 THR107 ARG108 PRO109 ASN110 ALA111 ILE113 GLN116 THR134 LEU135 ASP136 THR137 ARG139 HIS140 ASN159 HIS180 PHE206 SER207 GLU208 ILE211 ARG215 CYS264 SER265 THR266 ASP267 TRP289 GLY290 ARG310
Linalyl acetate	−6.7	PRO74 GLN77 SER79 TYR81 TRP82 THR107 ARG108 PRO109 ASN110 ALA111 ILE113 GLN116 THR134 LEU135 ASP136 THR137 ARG139 HIS140 ASN159 PHE206 SER207 GLU208 ILE211 ARG215 CYS264 SER265 THR266 ASP267 TRP289 GLY290 ARG310
DMSO	−3.1	PRO74 GLN77 SER79 TYR81 TRP82 ARG108 PRO109 ASN110 GLN116 THR134 LEU135 ASP136 THR137 ARG139 HIS140 GLN158 ASN159 HIS180 PHE206 SER207 ILE211 ARG215 SER265 THR266 ASP267 TRP289 GLY290 ARG310
